# Lignans: Quantitative Analysis of the Research Literature

**DOI:** 10.3389/fphar.2020.00037

**Published:** 2020-02-07

**Authors:** Andy Wai Kan Yeung, Nikolay T. Tzvetkov, Aneliya A. Balacheva, Maya G. Georgieva, Ren-You Gan, Artur Jozwik, Bożena Pyzel, Jarosław O. Horbańczuk, Ettore Novellino, Alessandra Durazzo, Massimo Lucarini, Emanuela Camilli, Eliana B. Souto, Atanas G. Atanasov, Antonello Santini

**Affiliations:** ^1^Oral and Maxillofacial Radiology, Applied Oral Sciences and Community Dental Care, Faculty of Dentistry, The University of Hong Kong, Hong Kong, China; ^2^Department of Biochemical Pharmacology and Drug Design, Institute of Molecular Biology “Roumen Tsanev”, Bulgarian Academy of Sciences, Sofia, Bulgaria; ^3^Pharmaceutical Institute, University of Bonn, Bonn, Germany; ^4^Institute of Urban Agriculture, Chinese Academy of Agricultural Sciences, Chengdu, China; ^5^The Institute of Genetics and Animal Breeding, Polish Academy of Sciences, Magdalenka, Poland; ^6^Department of Pharmacy, University of Napoli Federico II, Napoli, Italy; ^7^CREA-Research Centre for Food and Nutrition, Rome, Italy; ^8^Department of Pharmaceutical Technology, Faculty of Pharmacy, University of Coimbra (FFUC), Polo das Ciências da Saúde, Azinhaga de Santa Comba, Coimbra, Portugal; ^9^CEB-Centre of Biological Engineering, University of Minho, Campus de Gualtar, Braga, Portugal; ^10^Institute of Neurobiology, Bulgarian Academy of Sciences, Sofia, Bulgaria; ^11^Department of Pharmacognosy, University of Vienna, Vienna, Austria; ^12^Ludwig Boltzmann Institute for Digital Health and Patient Safety, Medical University of Vienna, Spitalgasse, Vienna, Austria

**Keywords:** lignans, pharmacology, chemistry, plant science, cancer, citation analysis, VOSviewer, Web of Science

## Abstract

The current study provides a comprehensive overview and analysis of the lignan literature. Data for the current study were extracted from the electronic Web of Science Core Collection database *via* the search string TOPIC = (“lignan*”) and processed by the VOSviewer software. The search yielded 10,742 publications. The ratio of original articles to reviews was 14.6:1. Over 80% of the analyzed papers have been published since the year 2000 and nearly 50% since the year 2010. Many of the publications were focused on pharmacology, chemistry, and plant sciences. The United States and Asian countries, such as China, Japan, South Korea, and India, were the most productive producers of lignan publications. Among the 5 most productive institutions was the University of Helsinki in Finland, the country that ranked 9^th^. Nineteen journals collectively published 3,607 lignan publications and were considered as core journals. Their impact factor did not correlate with the proportion of uncited papers. Highly cited publications usually mentioned phytoestrogen, isoflavone, daidzein, enterodiol, enterolactone, equol, genistein, and isoflavonoid. Cancer (e.g., breast cancer), cardiovascular disease, and antioxidation were the major themes. Clinical trials were estimated to contribute to 0.2–1.1% of the analyzed body of literature, so more of them should be conducted in the future to substantiate the beneficial effects and optimal dose of lignan intake in humans. Moreover, researchers can refer to these findings for future research directions and collaborations.

## Introduction

The current study aimed to perform a quantitative analysis on the literature of lignans to unveil the major contributors in terms of institutions, countries/regions, and journals. By analyzing the publication and citation data, the major research themes present in the lignan literature were identified and further discussed.

Lignans are 1,4-diarylbutan compounds derived from the shikimic acid biosynthetic pathway (Lewis and Davin, 1999; Imai et al., 2006). In the 1970s, it was still commonly believed that lignans were synthesized in plants only (Hartwell, 1976). It was only in the 1980s when scientists identified lignans produced by microbes living in humans and animals (Axelson et al., 1982). Geographically, the intakes are greater in the European population relative to the Asian population (Bhakta et al., 2006). The main common dietary lignans are secoisolariciresinol, lariciresinol, matairesinol, pinoresinol, medioresinol, and syringaresinol (Durazzo et al., 2018); the range of components is very wide and efforts on isolation of new compounds are being carried out (Eklund and Raitanen, 2019; Xiao et al., 2019). Plant lignans are metabolized to enterodiol and enterolactone, called enterolignans or mammalian lignans (Landete, 2012).

The recent work of Durazzo et al. (2018) well summarized the occurrence of lignans in food groups and existing lignan databases at European level. As reported by Durazzo et al. (2018), the main sources of dietary lignans are oilseeds such as flax, soy, rapeseed, and sesame; whole-grain cereals such as wheat, oats, rye, and barley; legumes; various vegetables and fruits (particularly berries); beverages (i.e., coffee, tea, and wine); and, recently, lignans are also determined in dairy products, meat, and fish (Valsta et al., 2003; Milder et al., 2005a; Milder et al., 2005b; Peñalvo et al., 2005; Thompson et al., 2006; Penalvo et al., 2007; Kuhnle et al., 2008a; Kuhnle et al., 2008b; Durazzo et al., 2009; Kuhnle et al., 2009a; Kuhnle et al., 2009b; Smeds et al., 2009; Moreno-Franco et al., 2011; Smeds et al., 2012; Durazzo et al., 2013a; Durazzo et al., 2013b; Mulligan et al., 2013; Durazzo et al., 2014b; Turfani et al., 2017; Angeloni et al., 2018; Angeloni et al., 2019).

Within the bioactive compounds realm (Santini et al., 2018; Santini and Novellino, 2018; Daliu et al., 2019; Durazzo et al., 2019), the class of lignans is of interest for their potential biological activities, i.e., estrogenic and antiestrogenic, antioxidant, anti-inflammatory, metabolism-modulating, anti-proliferative, and anticancerogenic properties (Baumgartner et al., 2011; Teponno et al., 2016; Wang et al., 2016; Linder et al., 2019; Zálešák et al., 2019). Moreover, it is worth mentioning that the spectrum of biological activities attributed to lignans is being enlarged, i.e., related to newly discovered compounds belonged to this group (Zhang et al., 2014; Gnabre et al., 2015; Su and Wink, 2015; Hongthong et al., 2016; Azam et al., 2019; Zhuang et al., 2019).

Several studies showed that consumption of lignan-rich diets, which contain vegetables, fruits, and whole grain products, may protect against chronic diseases, particularly hormone-dependent cancer and cardiovascular diseases (Ward et al., 2009; Peterson et al., 2010; Buck et al., 2011; Guglielmini et al., 2012; Penalvo and López-Romero, 2012; Zamora-Ros et al., 2012; Lowcock et al., 2013; Durazzo et al., 2014a; Rodríguez-García et al., 2019). Proper evaluation of adherence, efficacy, and communication aspects should be taken into account as well as the retrospective analysis of databases as per recent studies in the field (Iolascon et al., 2016; Scala et al., 2016; Guerriero et al., 2017; Menditto et al., 2018).

The overview presented in the current study should be helpful to readers in better understanding the lignan research community, identifying potential research directions and collaboration partners, and conducting more in-depth literature searches of chemicals/chemical classes of interest.

## Materials and Methods

In July 2019, we queried the Web of Science (WoS) Core Collection online database, owned by Clarivate Analytics, to identify lignan publications with the following search string: TOPIC = (“lignan*”). This search identified publications mentioning the word “lignan” or its derivatives in the title, abstract, or keywords. No additional filters were placed on the search.

### Data Extraction

Several aspects of each publication identified from the search were recorded, namely: (1) publication year; (2) institutions; (3) countries/regions of the institutions; (4) journal title; (5) WoS journal category; (6) type of publication; (7) language; and (8) number of total citations received. By using the “Export Records to File” function of WoS, full records and cited references of the identified publications were exported as “tab-delimited text files” to VOSviewer for additional processing.

The VOSviewer software (v.1.6.11, 2019) was used to analyze the titles and abstracts of publications, by breaking down the paragraphs into words and phrases, associating them with the citation data of the publications, and presenting the results in the form of a bubble map (Van Eck and Waltman, 2009). Default parameters were used for the analyses and visualizations. The size of a bubble represents the frequency of appearance of a term (multiple appearances of a word counted once, single use of the same word in a paper equally weighted). Two bubbles are positioned more closely to each other if the terms co-appeared more often in the analyzed publications. The color represents the averaged citations per publication (CPP). To simplify the bubble map, we analyzed and visualized words that appeared in at least 1% (n = 108) of the publications.

Apart from analyzing the whole dataset, we additionally probed into the articles published by the most prolific journals to see how many of them were uncited. According to Bradford's law of scattering, the core journals for a body of literature are defined as the prolific journals that collectively published 1/3 of the papers (Vickery, 1948). Using the current analyzed dataset, we tested if the core journals had their impact factor negatively correlated to the proportion of uncited papers, which was previously demonstrated in another field (Yeung, 2019). Pearson's correlation test was performed using SPSS 25.0 (IBM, New York, USA). Test results were significant if p < 0.05.

## Results

The literature search resulted in 10,742 publications. The earliest publications on lignans indexed in WoS were published in 1970, which isolated new lignans at that time and identified their structures (Corrie et al., 1970). Over 80% of the analyzed papers have been published since the year 2000, and nearly 50% since the year 2010. The numbers of original articles (*n* = 9,422) and reviews (*n* = 644) were in the ratio of 14.6:1. Reviews were more cited (CPP = 56.8) than original articles (CPP = 22.5). The majority of the publications were written in English (*n* = 10,483; 97.6%). Contributions came from 4,748 institutions located in 141 countries/regions and were published in 1,509 journals. The top five contributors with regard to WoS category, journal, institution, and country/region are listed in **Table 1**. It is worth mentioning that *Molecules* was the 6^th^ most productive journal, with 208 lignan publications (1.9%) and CPP of 11.9. Nineteen journals collectively published 3,607 lignan publications and were considered as core journals (**Table 2**). Their impact factor did not correlate with the proportion of uncited papers (r = -0.257, p = 0.289). Though University of Helsinki was among the top 5 most productive institutions, Finland was ranked 9^th^ in terms of countries/regions (n = 437, 4.1%). The 5 most productive countries were all from Asia, except the United States.

**Table 1:** The top five contributors in terms of Web of Science category, journal, institution, and country/region of publications concerning lignans.

Contributor	Publication count (% of total)	Citation per manuscript
*Web of Science category*		
Pharmacology pharmacy	2,522 (23.5%)	20.5
Chemistry medicinal	2,520 (23.5%)	18.9
Plant sciences	2,050 (19.1%)	22.9
Biochemistry molecular biology	1,796 (16.7%)	24.8
Chemistry multidisciplinary	1,509 (14.0%)	16.3
*Journal*		
Phytochemistry	587 (5.5%)	31
Journal of Natural Products	345 (3.2%)	27.1
Planta Medica	332 (3.1%)	18.9
Chemical and Pharmaceutical Bulletin	266 (2.5%)	28.4
Journal of Agricultural and Food Chemistry	213 (2.0%)	54.5
*Organization*		
Chinese Academy of Sciences	473 (4.4%)	13.8
University of Helsinki	246 (2.3%)	81.9
Chinese Academy of Medical Sciences Peking Union Medical College	195 (1.8%)	14.0
Kunming Institute of Botany	193 (1.8%)	12.9
Universidade de Sao Paulo	186 (1.7%)	18.1
*Country/Territory*		
China	2,482 (23.1%)	13.0
United States	1,321 (12.3%)	41.2
Japan	1,305 (12.1%)	24.5
South Korea	691 (6.4%)	15.5
India	638 (5.9%)	16.5

**Table 2:** Core journals publishing lignan papers.

Journal	Impact factor	Proportion of uncited lignan papers (in %)
Phytochemistry	2.905	1.5
Journal of Natural Products	4.257	1.4
Planta Medica	2.746	17.8
Chemical & Pharmaceutical Bulletin	1.405	0.8
Journal of Agricultural and Food Chemistry	3.571	3.8
Molecules	3.060	15.9
Tetrahedron Letters	2.259	1.1
Tetrahedron	2.379	1.8
Natural Product Research	1.999	13.7
Journal of Organic Chemistry	4.745	3.4
Fitoterapia	2.431	13.4
Biochemical Systematics and Ecology	1.127	12.1
Journal of Ethnopharmacology	3.414	2.5
Journal of Asian Natural Products Research	1.170	7.1
Bioorganic & Medicinal Chemistry Letters	2.448	2.8
Food Chemistry	5.399	4.8
Phytochemistry Letters	1.338	15.8
Bioscience, Biotechnology, and Biochemistry	1.297	3.2
Archives of Pharmacal Research	2.458	5.4

**Figure 1:**
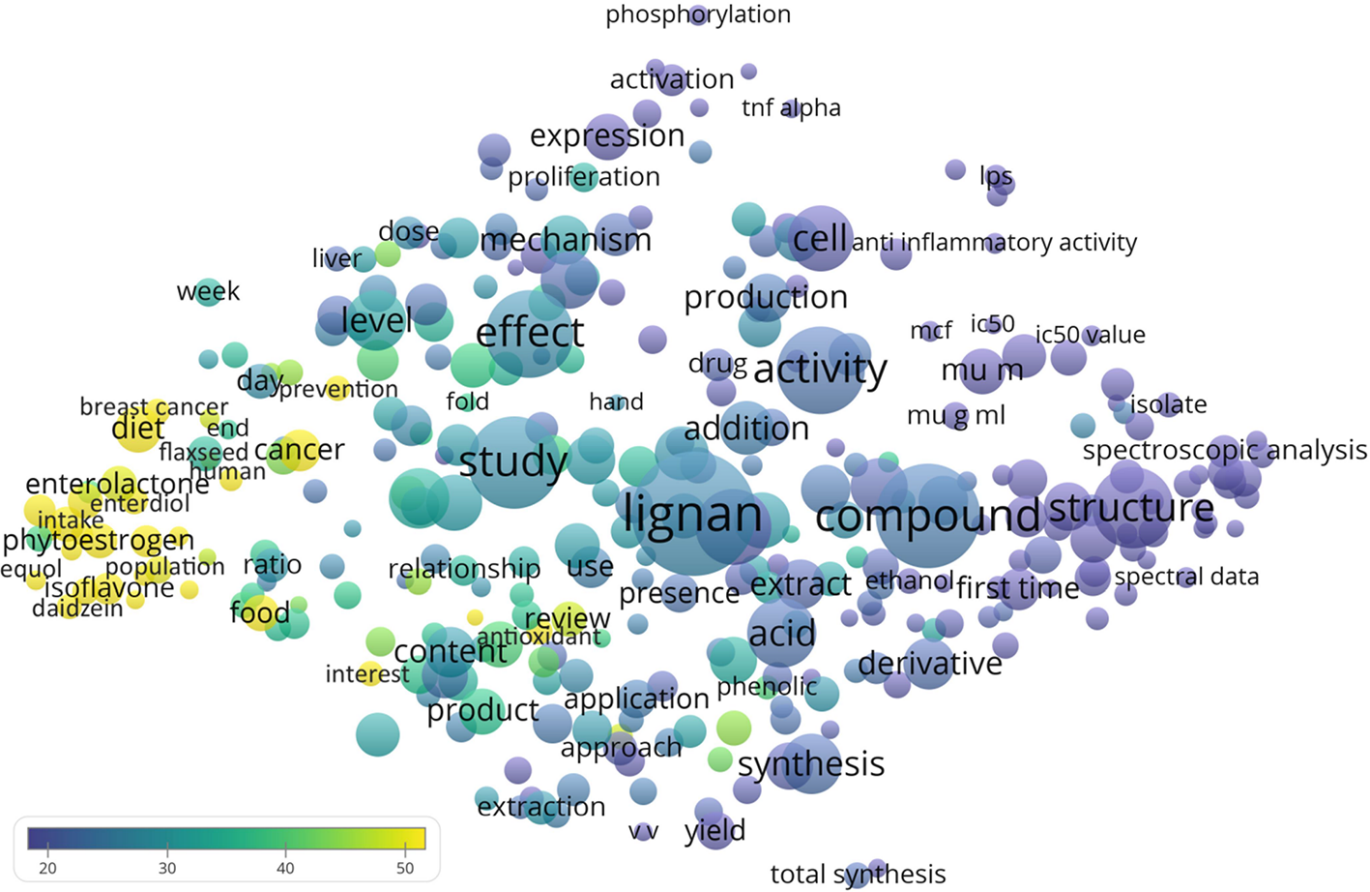
Bubble map visualizing words from titles and abstracts of the 10,742 lignan publications. VOSviewer software was used to evaluate the recurring terms. Only the 311 terms that appeared in at least 1% (n = 108) of the publications were analyzed and visualized. The size of a bubble represents the frequency of appearance of a term (multiple appearances within one publication were treated as one appearance). Two bubbles are positioned more closely to each other if the terms co-appeared more often. The color represents the averaged citations per publication.

**Figure 2:**
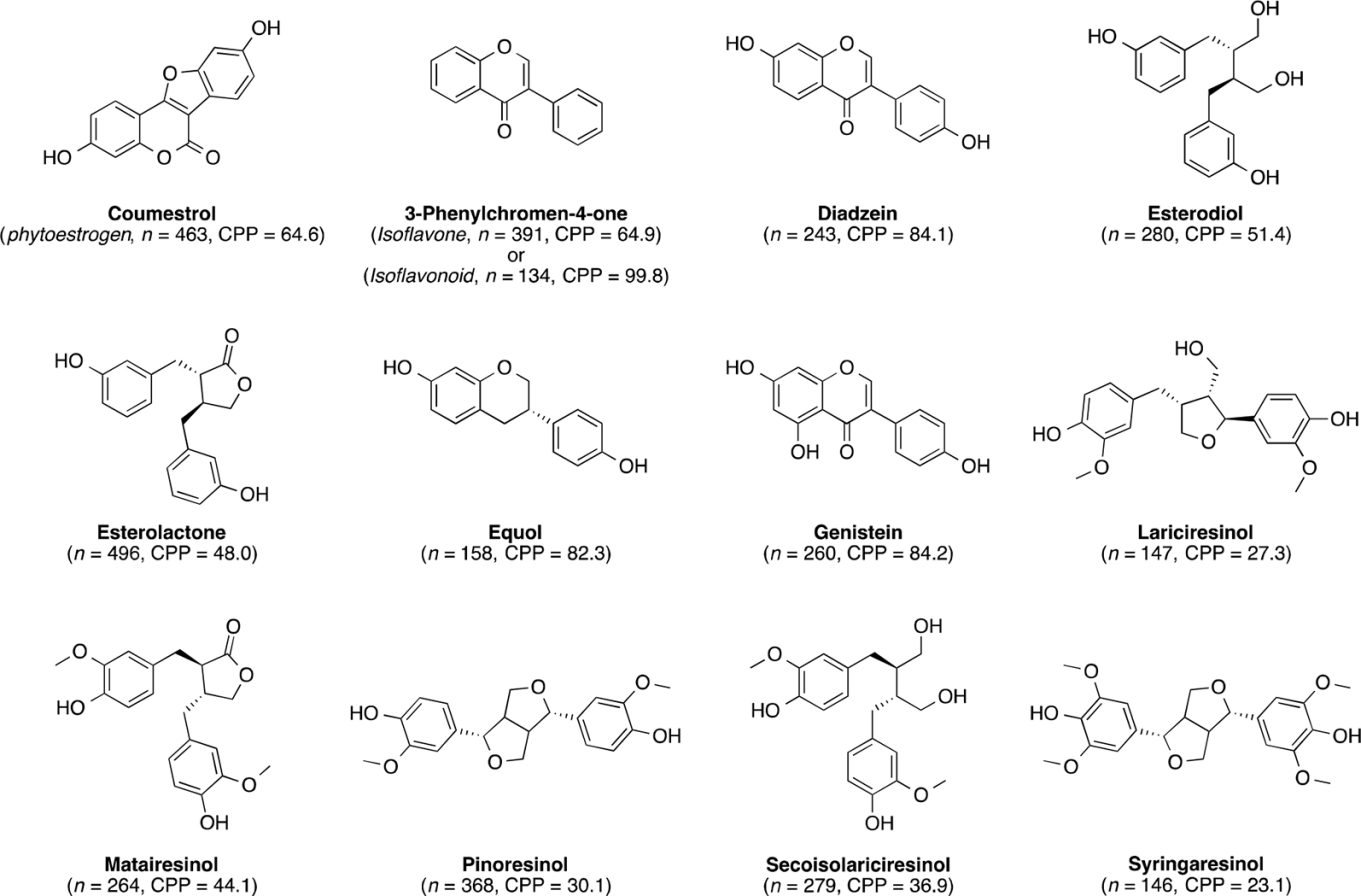
Chemical structures of key single phytochemicals or representatives of chemical classes that were often discussed in the evaluated lignan publications. In parentheses are the cited compound classes (*italic*), number of publications (*n*), and citations per publication (CPP) for each chemical or representative chemical class.

There were 311 terms that appeared in at least 1% (*n* = 108) of the 10,742 lignan publications (**Figure 1**). The highly cited publications usually mentioned phytoestrogen (4.3%, *n* = 463, CPP = 64.6), isoflavone (3.6%, n = 391, CPP = 64.9), or related terms such as daidzein (2.3%, n = 243, CPP = 84.1), enterodiol (2.6%, n = 280, CPP = 51.4), enterolactone (4.6%, n = 496, CPP = 48.0), equol (1.5%, n = 158, CPP = 82.3), genistein (2.4%, n = 260, CPP = 84.2), and isoflavonoid (1.2%, n = 134, CPP = 99.8). These terms were often mentioned together with cancer (6.1%, n = 655, CPP = 51.8), breast cancer (2.2%, n = 237, CPP = 51.8), or cardiovascular disease (1.5%, n = 157, CPP = 61.9). Some of the main common dietary lignans were frequently mentioned, such as lariciresinol (1.4%, n = 147; CPP = 27.3), matairesinol (2.5%, n = 264; CPP = 44.1), pinoresinol (3.4%, n = 368; CPP = 30.1), secoisolariciresinol (2.6%, n = 279; CPP = 36.9), syringaresinol (1.4%, n = 146; CPP = 23.1). The structures of these chemicals are shown in **Figure 2**. The top 20 recurring terms are listed in **Table 3**.

**Table 3:** The top 20 recurring terms from titles and abstracts.

Term	Appearance (% of 10,742 publications)
Lignan	5,700 (53.1%)
Compound	3,995 (37.2%)
Study	3,120 (29.0%)
Effect	2,878 (26.8%)
Activity	2,843 (26.5%)
Structure	2,443 (22.7%)
Analysis	2,121 (19.7%)
Acid	1,768 (16.5%)
Cell	1,629 (15.2%)
Level	1,367 (12.7%)
Concentration	1,365 (12.7%)
Synthesis	1,347 (12.5%)
Treatment	1,217 (11.3%)
Value	1,197 (11.1%)
Group	1,186 (11.0%)
Plant	1,144 (10.6%)
Addition	1,085 (10.1%)
Data	1,065 (9.9%)
Derivative	974 (9.1%)
Content	949 (8.8%)

The keywords listed by authors and WoS (KeyWords Plus) were collectively analyzed. There were 88 keywords that appeared in at least 1% (*n* = 108) of the lignan publications, and the 20 most common ones are listed in **Table 4**. The keywords suggested that antioxidation (4.3%) and apoptosis (3.2%) were two frequently investigated themes, and that *in vitro* (5.0%) studies were prevalent.

**Table 4:** The top 20 recurring keywords.

Keyword	Occurrence (% of 10,742 publications)
Lignans	3,825 (35.6%)
Lignan	1,358 (12.6%)
Constituents	1,217 (11.3%)
Derivatives	536 (5.0%)
Neolignans	536 (5.0%)
In-vitro	535 (5.0%)
Flavonoids	509 (4.7%)
Phytoestrogens	488 (4.5%)
Antioxidant activity	458 (4.3%)
Identification	438 (4.1%)
Cells	435 (4.0%)
Leaves	433 (4.0%)
Antioxidant	399 (3.7%)
Glycosides	388 (3.6%)
Flaxseed	383 (3.6%)
Inhibition	366 (3.4%)
Enterolactone	347 (3.2%)
Apoptosis	345 (3.2%)
Acid	344 (3.2%)
Expression	334 (3.1%)

To analyze the temporal changes in the keywords, we separately assessed lignan publications in three time periods: 1990s and before, 2000s, and 2010s. The top 20 recurring keywords for each of the three periods are listed in **Table 5**. Antioxidant activity rose to popularity since the 2000s. Apoptosis, cytotoxicity, and oxidative stress became popular in the 2010s.

**Table 5:** The top 20 recurring keywords in each decade.

1990s and before	Occurrence (% of 2,144)	2000s	Occurrence (% of 3,312)	2010s	Occurrence (% of 5,295)
Lignans	593 (27.7)	Lignans	1,304 (39.4)	Lignans	1,928 (36.4)
Lignan	192 (9.0)	Lignan	467 (14.1)	Constituents	708 (13.4)
Constituents	148 (6.9)	Constituents	361 (10.9)	Lignan	699 (13.2)
Neolignans	108 (5.0)	Phytoestrogens	254 (7.7)	In-vitro	366 (6.9)
Phytoestrogens	96 (4.5)	Neolignans	180 (5.4)	Flavonoids	324 (6.1)
Genistein	86 (4.0)	Derivatives	179 (5.4)	Antioxidant activity	313 (5.9)
Breast-cancer	60 (2.8)	Enterolactone	157 (4.7)	Antioxidant	307 (5.8)
Derivatives	60 (2.8)	Flaxseed	152 (4.6)	Cells	298 (5.6)
Identification	57 (2.7)	In-vitro	143 (4.3)	Derivatives	297 (5.6)
Cancer	50 (2.3)	Antioxidant activity	140 (4.2)	Leaves	295 (5.6)
Women	48 (2.2)	Flavonoids	138 (4.2)	Apoptosis	272 (5.1)
Chemistry	47 (2.2)	Phyto-estrogens	132 (4.0)	Identification	266 (5.0)
Flavonoids	47 (2.2)	Breast-cancer	127 (3.8)	Expression	253 (4.8)
Bark	46 (2.1)	Inhibition	119 (3.6)	Glycosides	252 (4.8)
Acid	45 (2.1)	Metabolism	119 (3.6)	Neolignans	248 (4.7)
Inhibition	44 (2.1)	Identification	115 (3.5)	Phenolic-compounds	210 (4.0)
Podophyllotoxin	44 (2.1)	Acid	114 (3.4)	Oxidative stress	209 (3.9)
Diet	42 (2.0)	Mammalian lignans	114 (3.4)	Flaxseed	203 (3.8)
Estrogens	42 (2.0)	Cells	113 (3.4)	Inhibition	203 (3.8)
Route	42 (2.0)	Podophyllotoxin	113 (3.4)	Cytotoxicity	194 (3.7)

## Discussion

The current literature analysis on lignan publications revealed the large publication shares from Asian countries, which were consistent with related bodies of literature such as antioxidants and curcumin (Yeung et al., 2019b; Yeung et al., 2019c). Examples of some highly cited original research papers recently published by Asian teams in the 2010s, without international collaborations, are discussed here. For instance, a Chinese paper reported results from sesame transcriptomes that provide useful information for understanding the relevant lignan biosynthesis molecular mechanism (Wei et al., 2011). Another Chinese team tested the effects of new lignans and neolignans on inhibiting nitric oxide production in mouse macrophages and against serum deprivation-induced PC12 cell damage (Xiong et al., 2011). These papers received over 100 citations. Meanwhile, Korean teams published the anti-inflammatory effects of several lignans isolated from *Schiandra chinensis* (Oh et al., 2010), and the hepatoprotective effect of pinoresinol isolated from *Forsythiae Fructus* (Kim et al., 2010). These papers were cited over 50 times. In Japan, a randomized controlled trial was conducted, and results found that oral intake of flaxseed (*Linum usitatissimum* L.) lignan could lower blood cholesterol level and risk of hepatic diseases in hypercholesterolemic men (Fukumitsu et al., 2010). Another Japanese team described an efficient synthetic route to synthesize herbindoles as naturally occurring forms (Saito et al., 2012). In India, researchers extracted, separated, and characterized sesame oil lignan (Reshma et al., 2010) and reported a phylogenetic analysis of *L. usitatissimum* L. (Barvkar et al., 2012). These Japanese and Indian papers had around 40 citations each. All these examples demonstrate the variety of the lignan research field, which ranged from basic sciences to human clinical trials.

Similar to the related research fields of berries, dietary natural products, and functional foods (Yeung et al., 2018a; Yeung et al., 2018b; Yeung et al., 2019d), the bubble map suggested that cancer and cardiovascular diseases were highly cited topics for lignan research. Readers can refer to comprehensive reviews on the relationship between phytoestrogens (such as lignans and isoflavonoids) and Western diseases (such as breast cancer and coronary heart disease) (Adlercreutz and Mazur, 1997; Rietjens et al., 2017). Their modulatory effects on steroid biosynthetic enzymes, hormone concentrations, and cellular events seem to be beneficial against cancer development (Adlercreutz and Mazur, 1997; Rietjens et al., 2017). In the early 1990s, a Finnish-Japanese collaboration probed into the low mortality in hormone-dependent cancer among the Japanese and found that they had high intake of soybean products rich in phytoestrogens, as demonstrated by a high concentration of isoflavonoids (and lignans to a lesser extent) excreted in their urine (Adlercreutz et al., 1991). In the year 1997, a case-control study published in *Lancet* reported that a high intake of phytoestrogens particularly lignan enterolactone and isoflavone equol could substantially reduce breast cancer risk in women (Ingram et al., 1997). Later, another paper reviewed data on existing epidemiologic studies and suggested that lignans and flavonoids have beneficial effects on cardiovascular diseases and lung cancer, but not other cancers (Arts and Hollman, 2005). The issues of low bioavailability might partly explain the differences in the results obtained between studies using cell/animal models and humans, particularly for the anti-cancer effects (Yang et al., 2001).

In addition, the bubble map can also relate to some of the potential biological activities of lignans, e.g., estrogenic and antiestrogenic, antioxidant, anti-inflammatory, and anticancerogenic properties (Baumgartner et al., 2011; Teponno et al., 2016; Wang et al., 2016; Linder et al., 2019; Zálešák et al., 2019), especially with antioxidant and anti-inflammatory activity being identified as frequently mentioned terms, whereas they were strong interests in phytoestrogen and cancer.

By limiting to “articles” (excluding other publication types such as reviews), a quick query of “clinical trial*” within the analyzed body of literature returned with 50 hits only. After evaluation, we found that there were only 19 randomized clinical trials, which was equivalent to 0.2% of the 10,742 lignan publications. A follow-up search in PubMed database with a query of “lignan*” and limited article type to “Clinical Trial” returned with 121 hits, which was equivalent to 1.1% of the analyzed publications. With such a small ratio of clinical trials in the lignan research literature, we believe that more clinical trials should be conducted to substantiate the beneficial effects and optimal dose of lignan intake on humans. In addition, researchers are currently experiencing common difficulties in estimating the dietary intakes of lignans (and also other non-nutritive substances) because they are not routinely included in the food composition tables, and there exists variability in contents reactive to soil quality, sun exposure, etc. All these complicate the works concerning the dose of lignan intake.

This study inherited some limitations, such as using indexed data based on a single database (WoS). Furthermore, the latest research trends, if any, might remain undetected due to a lack of time to accumulate publication and citation counts. Similar to previous literature analyses on curcumin and resveratrol (Yeung et al., 2019a; Yeung et al., 2019b), we did not analyze the authorship of the lignan publications, as there existed many Chinese authors with similar initials that caused inaccurate counting. Analyzing authorship by authors' full names was also not practical, as many publication records listed author initials only. Moreover, the analysis cannot evaluate the scientific methods used to determine the research findings (e.g., distinguish between *in vivo* work used to determine mechanistic relationships at a molecular level, and disease associations elucidated from population research). For an analysis of over 10,000 publications, this requires additional automatic labeling of the documents (data tagging), which is currently very limited in the literature databases. For Web of Science, for example, there are only a few publication types, e.g., articles, reviews, editorials. Besides, lignan sub-types and method of action in metabolizers are not analyzed.

Overall, the current report identified the terms and themes in the lignan research literature, being important in terms of publication and citation data. Results revealed several recurring or highly cited themes, implying that the bibliometric analysis was able to quantitatively highlight the topics in the field deemed important by the field experts.

## Conclusions

To summarize, a bibliometric analysis was conducted to evaluate publications on lignans. The current findings revealed that the United States and Asian countries, such as China, Japan, South Korea, and India, were the most productive countries. Some productive institutions were based outside these countries, such as the University of Helsinki in Finland. Many of the publications were focused on pharmacology (23.5%), chemistry (23.5%), and plant sciences (19.1%). Over 80% of the analyzed papers have been published since year 2000, and nearly 50% since year 2010. The highly cited publications usually mentioned specific terms such as phytoestrogen, isoflavone, daidzein, enterodiol, enterolactone, equol, genistein, isoflavonoid, cancer, breast cancer, or cardiovascular disease. Some frequently mentioned and discussed main common dietary lignans were lariciresinol, matairesinol, pinoresinol, secoisolariciresinol, and syringaresinol.

## Data Availability Statement

The datasets generated for this study are available on request to the corresponding authors.

## Author Contributions

AY, AD, AA, and AS conceived the work, performed data collection and analysis, and drafted the manuscript. All authors critically revised the manuscript and approved the submission of the manuscript.

## Funding

AA acknowledges the support by the Polish KNOW (Leading National Research Centre) Scientific Consortium “Healthy Animal—Safe Food,” decision of the Ministry of Science and Higher Education No. 05-1/KNOW2/2015. EN and AS acknowledge the support of the research project Nutraceutica come supporto nutrizionale nel paziente oncologico, CUP: B83D18000140007.

## Conflict of Interest

The authors declare that the research was conducted in the absence of any commercial or financial relationships that could be construed as a potential conflict of interest.
